# A study of Rose Bengal against a 2-keto-3-deoxy-d-*manno*-octulosonate cytidylyltransferase as an antibiotic candidate

**DOI:** 10.1080/14756366.2020.1751150

**Published:** 2020-06-26

**Authors:** Suwon Kim, Seri Jo, Mi-Sun Kim, Dong Hae Shin

**Affiliations:** Graduate School of Pharmaceutical Sciences, Ewha Woman’s University, Seoul, Republic of Korea

**Keywords:** CMP-2-keto-3-deoxy-d-*manno*-octulosonic acid biosynthesis pathway, 2-keto-3-deoxy-d-*manno*-octulosonate cytidylyltransferase (KdsB), melioidosis, multi-drug resistance, Rose Bengal

## Abstract

Frequent occurrences of multi-drug resistance of pathogenic Gram-negative bacteria threaten human beings. The CMP-2-keto-3-deoxy-d-*manno*-octulosonic acid biosynthesis pathway is one of the new targets for antibiotic design. 2-Keto-3-deoxy-d-*manno*-octulosonate cytidylyltransferase (KdsB) is the key enzyme in this pathway. KdsB proteins from *Burkholderia pseudomallei* (*Bp*), *B. thailandensis* (*Bt*), *Pseudomonas aeruginosa* (*Pa*), and *Chlamydia psittaci* (*Cp*) have been assayed to find inhibitors. Interestingly, Rose Bengal (4,5,6,7-tetrachloro-2′,4′,5′,7′-tetraiodofluorescein) was turned out to be an inhibitor of three KdsBs (*Bp*KdsB, *Bt*KdsB, and *Pa*KdsB) with promising IC_50_ values and increased thermostability. The inhibitory enzyme kinetics of Rose Bengal revealed that it is competitive with 2-keto-3-deoxy-*manno*-octulosonic acid (KDO) but non-competitive against cytidine 5′-triphosphate (CTP). Induced-fit docking analysis of *Pa*KdsB revealed that Arg160 and Arg185 together with other interactions in the substrate binding site seemed to play an important role in binding with Rose Bengal. We suggest that Rose Bengal can be used as the scaffold to develop potential antibiotics.

## Introduction

The 2-keto-3-deoxy-*manno*-octulosonic acid (KDO) which is an eight carbon acidic sugar composing the lipopolysaccharide (LPS) has a vital role in keeping outer membrane (OM) integrity and viability of most Gram-negative bacteria^1^^,^^2^. The KDO biosynthesis pathway consists of six enzymes: Ribose-5-phosphate isomerase (RpiA)^3^; d-arabinose-5-phosphate isomerase (KpsF)^4^; KDO-8-phosphate synthase (KdsA)^5^; KDO-8-phosphate phosphatase (YrbI)^6^; 2-keto-3-deoxy-d-*manno*-octulosonate cytidylyltransferase (KdsB), and glycosyltransferase (WaaA) which transfers the KDO from cytidine 5′-monophospho-2-keto-3-deoxy-*manno*-octulosonic acid (CMP-KDO) to the lipid A^7^. In LPS, KDO molecules are important to connect the core oligosaccharide with the lipid A^8–10^. Therefore, the KDO biosynthesis pathway has been considered as the potential target for the advanced antibiotics against Gram-negative bacteria^1^^,^^11^.

*Burkholderia pseudomallei* (*Bp*) is the etiologic agent of a serious and often fatal syndrome known as melioidosis^12–15^. Melioidosis features various symptoms like a self-limiting abscess, sepsis, necrotising pneumonia, osteomyelitis, and dissemination to the solid organs and brain^15–19^. On the other hand, *Burkholderia thailandensis* (*Bt*) which is highly relevant to *Bp*^20^ is reported to cause sporadic human disease even if *Bt* has been informed as non-pathogenic bacteria^21^^,^^22^. A similar but differently classified microbe, *Pseudomonas aeruginosa* (*Pa*), is an opportunistic pathogen and common Gram-negative bacillus in and around people^23^. Almost all infection cases of *Pa* can progress through nullifying host defence systems. *P. aeruginosa* infection can occur to patients who have general immunosuppression because of HIVs^24–26^. Also, *Pa* can cause various disease in our whole body, as secondary consequent results of the collapse of normal physiological function such as bacteraemia after severe burn, chronic lung infection in cystic fibrosis and acute ulcerative keratitis in soft contact lenses users^23^. In the same context, *Pa* is one of the major pathogens causing hospital-acquired infections^27^.

Three Gram-negative bacteria above have already been reported for their antibiotics resistance. Acquired or intrinsic resistant *Burkholderia* species for primary antibiotic treatment has been identified^28–30^. Moreover, *Pa* is resistant to an extensive range of antibiotics and can advance resistance to antimicrobials which are frequently used through mutations in genes or by the acquirement of resistant factors^27^. To overcome these resistant bacteria, we targeted KdsB involved in KDO biosynthesis from four pathogenic microbes (*Bp*, *Bt*, *Pa*, and *Chlamydia psittaci* (*Cp*)) to find potent antibiotic candidate molecules.

## Materials and methods

### Preparation of protein native KdsB proteins

The two of four KdsB genes (*BpkdsB* and *BtkdsB*) (NCBI reference sequence: NC_006350.1; NC_007651.1) coding for the proteins (*Bp*KdsB and *Bt*KdsB) were amplified by using of primers and ligated into the amplified expression vector pB_2_ by way of ligation-independent cloning (LIC) method. The rest of genes (*CpkdsB* and *PakdsB*) (NCBI reference sequence: WP_006343263.1; WP_003106922.1) coding for the proteins (*Cp*KdsB and *Pa*KdsB) were inserted in the pBT_7_ plasmid DNA (pDNA) and gained from Bioneer (Daejeon, South Korea). To express the proteins, we transformed pDNAs into *Escherichia coli* (*E. coli*) BL21 (DE3). Transformed *E. coli* cells were incubated on Luria-Bertani (LB) agar plates. Several colonies were chosen and grown in test tubes with a cap to determine the condition for culturing in bulk. In the process, a cell stock was prepared and frozen. The mass culture proceeded at 310 K with shaking. As the absorbance at 600 nm of broth reached 0.6–0.8, isopropyl-β-d-1-thiogalactopyranoside was put for expression of KdsB proteins (KdsBs). The cultures of *Bp*KdsB, *Bt*KdsB, and *Cp*KdsB were incubated at 298 K for 16 h and the culture of *Pa*KdsB was incubated at 310 K for 3 h. The expressed proteins contained non-cleavage N-terminal His_6_-tags followed by five glycines in *Bp*KdsB, *Bt*KdsB (MHHHHHH GGGGG) and eight amino acids in *Cp*KdsB, *Pa*KdsB EFSQQDSD (MHHHHHH EFSQQDSD). To collect cells, we centrifuged the culture fluid with a high-speed refrigerated centrifuge at 7650×*g* (6500 rev min^−1^) for 10 min at 277 K. The cultured cell pellet was suspended and fragmentised using a Digital Sonifier 450 (Branson Ultrasonics Co., Danbury, CT). Cell debris was pelleted by centrifugation. Using a HisTrap column (GE Healthcare, Piscataway, NJ), Affinity chromatography was done with the supernatant on an ÄKTA explorer system (GE Healthcare, Piscataway, NJ). Ion-exchange chromatography has been done as the secondary purifications using a 5 ml Hi-Trap Q column (GE Healthcare, Piscataway, NJ). The mobile phase buffer of the Q column and the concentration of sodium chloride at which proteins were eluted for each KdsB are organised in Table 1. The purified native proteins were concentrated to adequate concentration for using in the assay. The purity of proteins was at least 95% as considered by SDS/PAGE.

**Table 1.** The Q column experiments.

Protein	Size^a^ (kDa)	Buffer^b^	NaCl^c^ (M)
*Bp*KdsB	30	20 mM Tris–HCl pH 8.5	0.24
*Bt*KdsB	28.5	20 mM Tris–HCl pH 8.0	0.66
*Cp*KdsB	30.4	20 mM Tris–HCl pH 7.5	N/A^d^
*Pa*KdsB	29.5	20 mM Bis–Tris pH 7.0	0.27

^a^ The molecular weight of the proteins on SDS-PAGE.

^b^ The equilibration buffer conditions.

^c^ The NaCl concentration at elution.

^d^ *Cp*KdsB was done only with the affinity chromatography.

### Chemical screening with a malachite green assay method

The screening of about two hundred chemical compounds (Supplementary Table 1) was performed with a malachite green assay method which is a photometric method^31^. The principle of this method is: when KdsBs transfer the cytidine 5′-monophosphate (CMP) moiety from cytidine 5′-triphosphate (CTP) to KDO, CMP-KDO, and pyrophosphate (PPi) are produced. PPi was decomposed into two phosphates by inorganic pyrophosphatase (IPP) and phosphates were measured by the malachite green method. CTP and KDO purchased from Sigma (St. Louis, MO) were used as a real substrate. A colour reagent of the malachite green method for phosphate detection was a mixture of ammonium molybdate ((NH_4_)_6_Mo_7_O_24_), malachite green solution and Tween 20 in the ratio 1:3:0.1. The mixture was filtered through a 0.20 μm PVDF syringe filter (Younginfrontier Inc., Seoul, Republic of Korea) and allowed to stand at room temperature (RT) for 1 h before use. The probability of inhibitory function of each chemical was investigated by detecting the difference in absorbance between the reaction mixtures with and without KdsBs.

### Enzyme kinetics of KdsBs

The malachite green assay method^31^ was used to study steady-state kinetics of KdsBs (*Bp*KdsB, *Bt*KdsB, and *Pa*KdsB). For the kinetic studies, reaction mixtures including 5 mM Tris–HCl (pH 7.5), 10 mM MgCl_2_, 0.04 unit IPP, and 0.01 mg ml^−1^ KdsBs with different concentrations of CTP (0.00098–0.25 mM) at the constant concentration of KDO (0.25 mM) and with different concentrations of KDO (0.000195–1 mM) at the constant concentration of CTP (0.1 mM) were used. After incubation at RT for an hour, 40 μl of reaction mixtures were mixed with the malachite reagent (160 μl). The mixtures were left for 10 min to develop the colour. The standard curve was plotted and devices were used as in previous studies^32^.

### IC_50_ value of Rose Bengal

The malachite green assay method^31^ was also used to study the dose-dependent inhibitory effect of Rose Bengal (4,5,6,7-tetrachloro-2′,4′,5′,7′-tetraiodofluorescein) purchased from Tokyo Chemical Industry (Tokyo, Japan). The 40 μl of reaction mixtures containing 5 mM Tris–HCl (pH 7.5), 10 mM MgCl_2_, and 0.01 mg ml^−1^ KdsBs with different concentrations of Rose Bengal (0.098–100 μM) were incubated at RT for an hour. The reaction mixtures lacking KdsBs with the same concentration of Rose Bengal used as above were also incubated at RT for an hour and measured as blanks. The reaction was initiated by adding 0.25 mM KDO and 0.1 mM CTP and stood for an hour. After incubation, 160 μl of the same malachite reagent used above was added and left for 10 min. The absorbance was measured at 620 nm using the microplate spectrophotometer (Spectramax 190, Molecular Devices Corporation, Sunnyvale, CA). Rose Bengal is a photoreactive compound with its absorbance peaks at 258 nm, 519 nm, and 562 nm^33^. Therefore, the measurement wavelength of the malachite green assay is not influenced by Rose Bengal. The percentage reactivity (%Reactivity) was calculated depending on the difference in absorbance of whether or not KdsBs was present in the reaction mixture. The IC_50_ values of Rose Bengal against KdsBs were calculated by a nonlinear regression analysis using GraphPad Prism 8.3.0 (GraphPad Software, La Jolla, CA).

### Inhibitory enzyme kinetics

To specify the binding site of compounds for the induced fit docking, we studied the inhibitory enzyme kinetics using malachite green assay. Reaction mixtures including 5 mM Tris–HCl (pH 7.5), 10 mM MgCl_2_, 0.04 unit IPP, 0.01 mg ml^−1^ *Pa*KdsB, and 14.66 µM Rose Bengal with different concentrations of CTP (0.00098–0.25 mM) at the constant concentration of KDO (0.25 mM) and with different concentrations of KDO (0.000195–2.5 mM) at the constant concentration of CTP (0.1 mM) were used.

### Sample preparation and circular dichroism (CD) spectroscopy

Native *Bp*KdsB (0.2 mg ml^−1^) and *Bp*KdsB co-incubated at 4 °C with Rose Bengal in a mole ratio of 1:20 (protein:Rose Bengal) were ready for *T*_m_ value measurement. The CD sample buffer conditions were 20 mM Tris pH 8.5. CD measurements were taken with a J-1500 Circular Dichroism Spectrophotometer (JASCO Co., Mary’s Court, Easton, MD) equipped with a computer-controlled water bath, and samples were placed in a Rectangular Quartz Cell of 1 mm optical path length. The thermal denaturation was observed at 222 nm from 25 °C to 95 °C at 1 °C min^−1^ rate. Data analysis and display were done using Spectra Manager Version 2, Thermal Denaturation Multi Analysis version 2.1.0.1 software.

To ascertain the thermostability of KdsBs with Rose Bengal, three native KdsBs (*Bp*KdsB, *Bt*KdsB, and *Pa*KdsB) (1 mg ml^−1^) and KdsBs bound with Rose Bengal (0.625 mM for *Bp*KdsB, 1.25 mM for *Bt*KdsB, and 2.5 mM for *Pa*KdsB) were prepared. All samples were heated at 80 °C for 5 min and Rose Bengal bound samples were also heated at 100 °C for 5 min.

### Ligand preparation, target preparation, and induced-fit docking

All the docking and scoring calculations were performed using the Schrödinger software suite (Maestro, version 11.8.012) against the substrate and nucleotide binding sites of *Pa*KdsB. The SDF file of Rose Bengal was got from the PubChem database. The file was imported into Maestro and prepared for docking using ligand preparation. The atomic coordinates of the crystal structure of *Pa*KdsB (PDB ID: 4XWI) were saved from the Protein Data Bank and prepared by removing all solvent and adding hydrogens and minimal minimisation using Protein Preparation Wizard. Ioniser was used to generate an ionised state of Rose Bengal at the target pH 7.5 ± 2.0. The input for an induced-fit docking is the prepared low-energy ligand forms. The induced-fit docking protocol^34^ was worked on the graphical user interface, Maestro 11.8.012 linked with the Schrödinger software. Receptor sampling and refinement were conducted for residues within 5.0 Å of each ligand for each ligand–protein complex. With Prime^35^, a side‐chain sampling and prediction module, as well as the backbone of the target protein, were minimised in energy. A total of induced‐fit receptor conformations were generated for Rose Bengal. Finally, the ligand poses were scored using a combination of Prime and Glide Score scoring functions^36^.

## Results

### Chemical screening with a malachite green assay method

The enzyme activity of KdsBs was confirmed and the chemical screening of about 200 compounds was done with the malachite green assay method^31^. It is a fast, reproducible, colorimetric method for measuring inorganic free phosphate in aqueous solutions. Hence, the assay offered great ease for exploring potent inhibitors against KdsBs. A list of compounds’ names is provided as Supplementary Table 1 in the Supplementary Materials. As a result of the screening, one potent inhibitor, Rose Bengal, blocking three KdsBs (*Bp*KdsB, *Bt*KdsB, and *Pa*KdsB) was detected. However, no hit was found against *Cp*KdsB.

### Enzyme kinetics of three KdsBs

**Figure 1.**
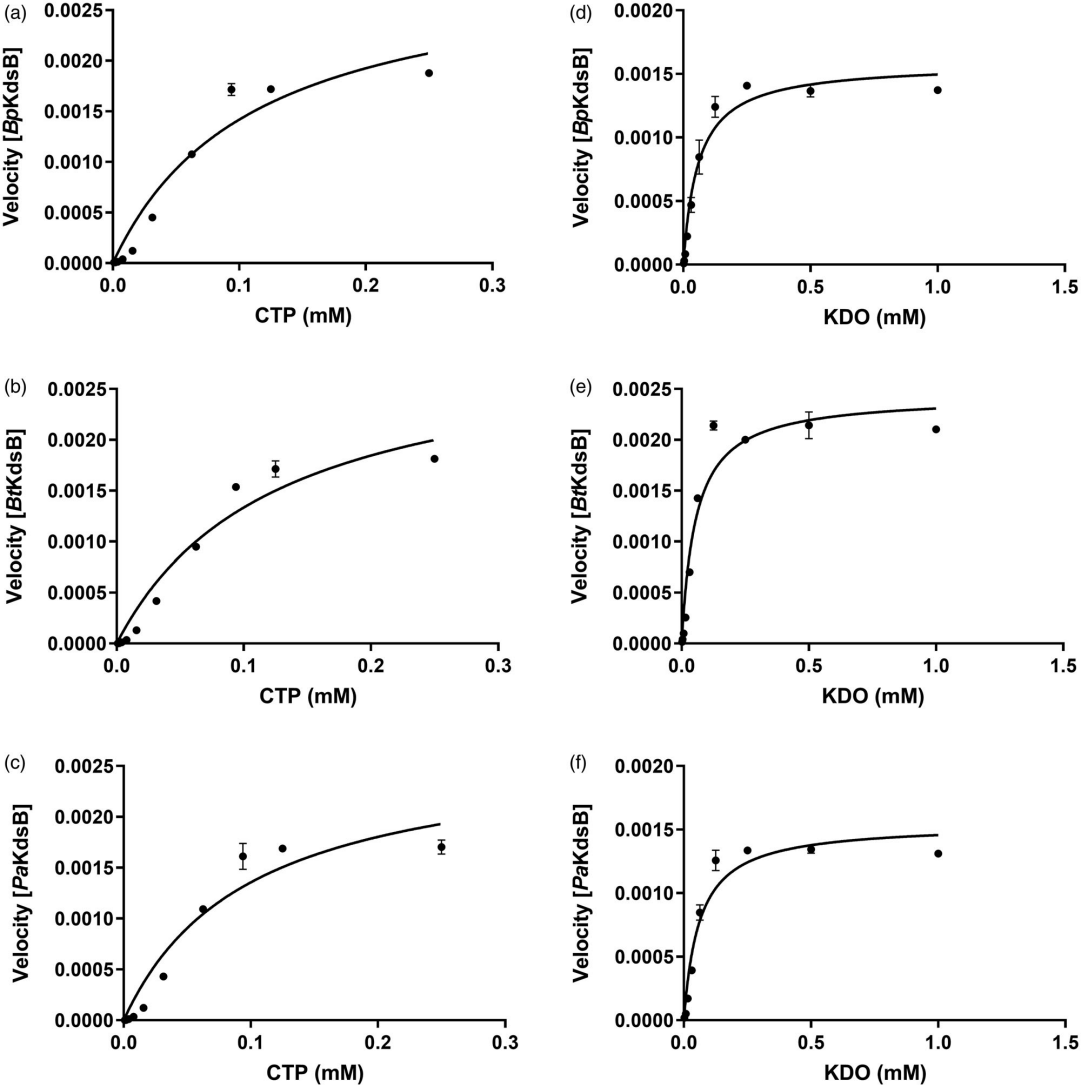
The specific enzyme activities of *Bp*KdsB, *Bt*KdsB, and *Pa*KdsB. The enzyme activities with (a–c) different concentrations of CTP in the presence of 0.25 mM KDO and (d–f) different concentrations of KDO in the presence of 0.1 mM CTP.

To determine the kinetic parameters for KDO and CTP, enzyme assays were performed with various concentrations of CTP at a constant KDO concentration (0.25 mM) and with various concentrations of KDO at a constant CTP concentration (0.1 mM). The graphs of the velocities versus concentrations of KDO and CTP are shown in Figure 1. The graphs indicated that the maximum velocity was obtained at 0.1 mM CTP in the presence of 0.25 mM KDO and at 0.25 mM KDO in the presence of 0.1 mM CTP. The kinetic parameter data of CTP and KDO at the steady-state are organised in Table 2.

**Table 2.** The KdsBs kinetics parameters.

KdsBs	CTP		KDO	
	*k*_cat_ (min^–1^)	*K*_m_ (mM)	*k*_cat_ (min^–1^)	*K*_m_ (mM)
*Bp*KdsB	9.986 ± 1.322	0.1111 ± 0.031	5.278 ± 0.223	0.059 ± 0.009
*Bt*KdsB	8.540 ± 1.156	0.1239 ± 0.034	6.961 ± 0.372	0.056 ± 0.011
*Pa*KdsB	7.911 ± 1.104	0.0983 ± 0.030	4.547 ± 0.230	0.061 ± 0.011

### The IC_50_ value of Rose Bengal

**Figure 2.**
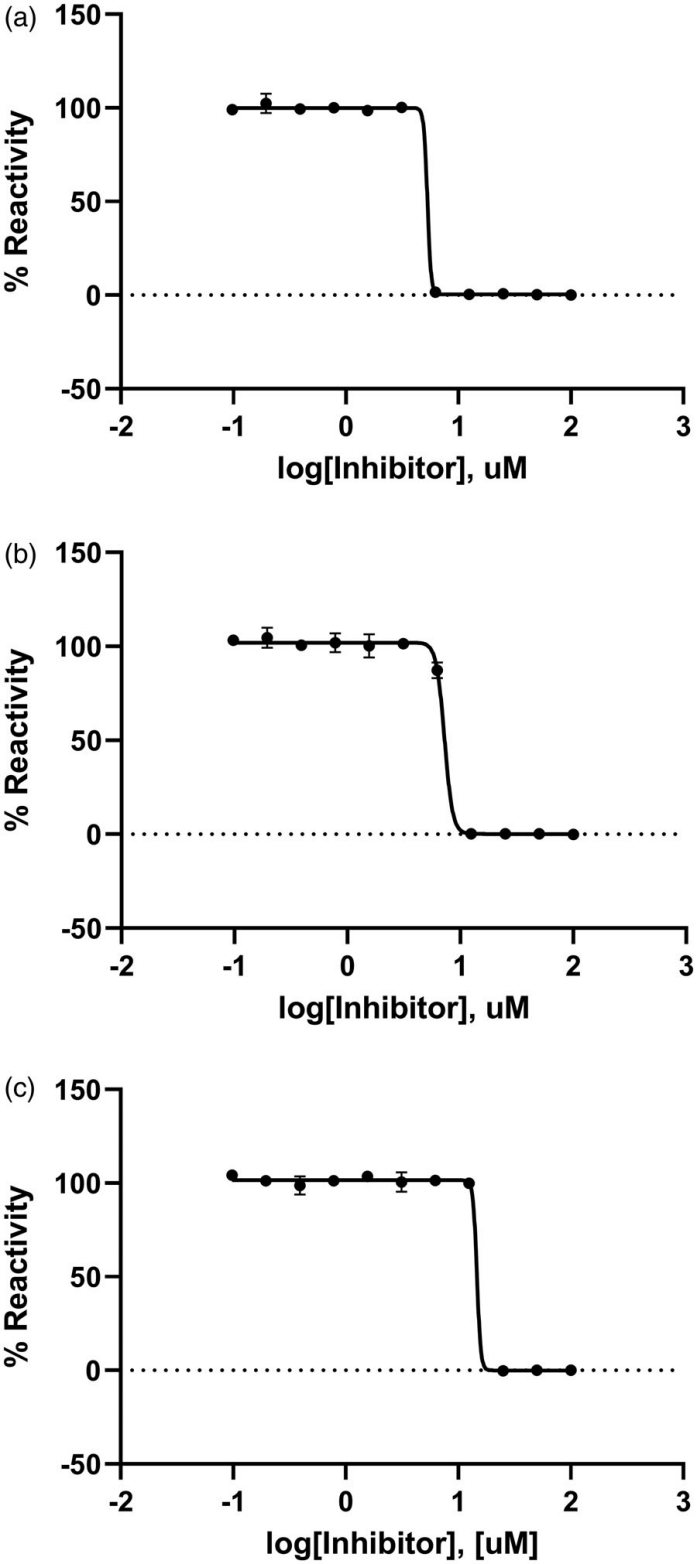
A dose-dependent inhibitory curve for the inhibition of (a) *Bp*KdsB, (b) *Bt*KdsB, and (c) *Pa*KdsB by Rose Bengal.

The inhibitory effect of Rose Bengal against KdsBs was found through a chemical screening with the malachite green assay method. The dose-dependent inhibitory effect of Rose Bengal was estimated at the saturated substrate concentrations (0.1 mM CTP and 0.25 mM KDO). The results were plotted as log inhibitor concentration versus percentage reactivity of absorbance (Figure 2). The IC_50_ value determined from the dose-dependent inhibitory curve of Rose Bengal: *Bp*KdsB was 5.31 µM; *Bt*KdsB was 7.24 µM; *Pa*KdsB was 14.66 µM.

### *T*_m_ value CD-ORD and thermostability

The binding effect of Rose Bengal was tested with the measurement of the *T*_m_ values of native and complex *Bp*KdsB. The *T*_m_ value of native *Bp*KdsB was 76.8 °C. Interestingly, that of Rose Bengal bound form was not measurable because *Bp*KdsB became heat stable and thus did not denatured at 100 °C. The similar effect on thermostability increased by Rose Bengal was also found in *Bt*KdsB and *Pa*KdsB. The thermal denaturation test showed that three native KdsBs were all precipitated at around 80 °C. However, the three KdsBs treated with Rose Bengal with their full inhibitory concentration were all stable at 100 °C for 5 min. Therefore, the increased thermostability of KdsBs could be the sign of compound binding.

### Inhibitory enzyme kinetics and docking

**Figure 3.**
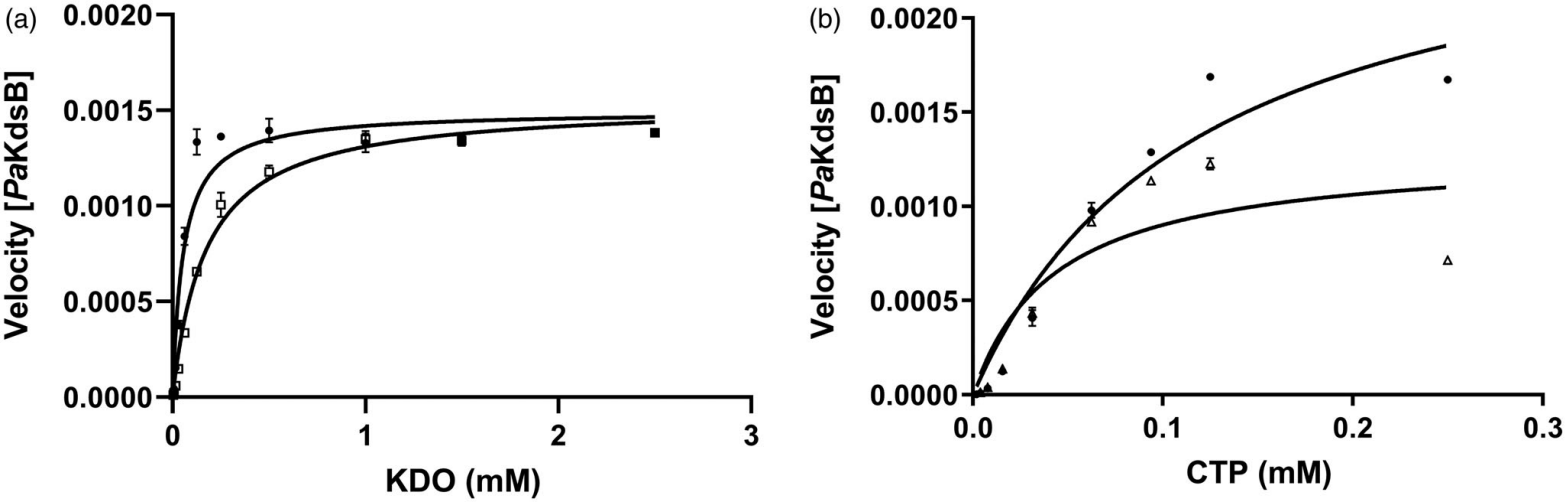
The inhibitory enzyme kinetics of KdsB from *Pseudomonas aeruginosa* (*Pa*KdsB). The enzyme activities with (a) different concentrations of CTP in the presence of 0.25 mM KDO and (b) different concentrations of KDO in the presence of 0.1 mM CTP. Filled circles represent the inhibitory enzyme kinetics with DMSO and squares for with 14.66 µM Rose Bengal in (a). Filled circles represent the inhibitory enzyme kinetics with DMSO and triangles for with 14.66 µM Rose Bengal in (b).

**Figure 4.**
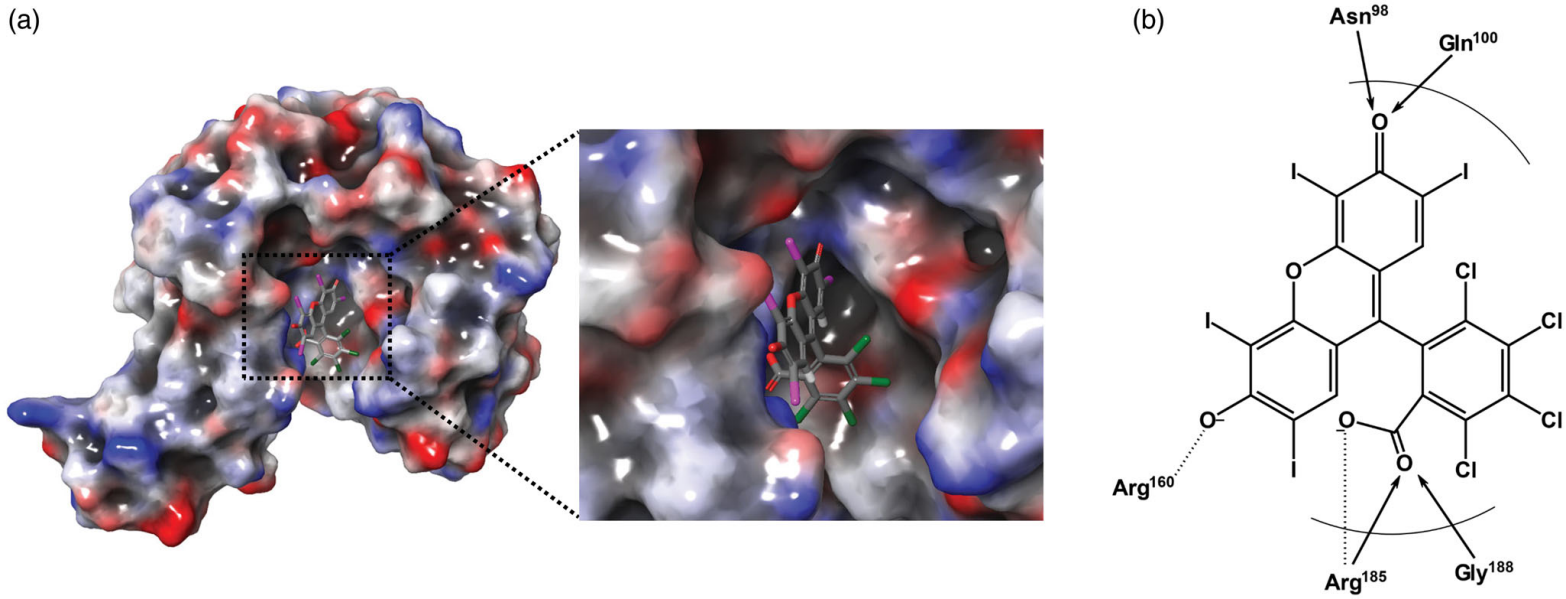
A predicted docking mode of Rose Bengal in the catalytic site of *Pa*KdsB. (a) The docking pose of Rose Bengal was depicted on the electrostatic surface potential of *Pa*KdsB (red, negative; blue, positive; white, uncharged). The enlarged view of the active site docked with Rose Bengal was depicted with dot lines. In addition, (b) a 2D schematic representation of the docked Rose Bengal with *Pa*KdsB was drawn. Figures were created with Maestro v11.5.011. The dot line represents the ion interaction by Arg160 and Arg185 and the arrow represents the hydrogen bond interaction with Asn95, Gln100, and Gly188.

According to the inhibitory enzyme kinetics, Rose Bengal was turned out to be a competitive inhibitor against KDO in *Pa*KdsB (Figure 3). To deduce the binding modes of Rose Bengal with KdsBs in the atomic level, an in-depth theoretical investigation with an induced-fit docking study using the Schrödinger programme was carried out. The crystal structure of *Pa*KdsB deposited in the Protein Data Bank was retrieved and docked with Rose Bengal to predict its binding mode. Top-ranked structures with the highest Glide *g*-scores from the induced-fit docking results were surveyed. The top model was obtained among docked models in the substrate binding pocket and had the *g*-score of –7.19. The *g*-score of the best model docked in the nucleotide binding site was –5.62. The predicted complex structure and 2D schematic representation of the top model are illustrated in Figure 4.

## Discussion

All of the four KdsBs from pathogenic bacteria were well expressed. The sequence alignment showed that their overall sequence identities against *Pa* were roughly from 38 to 55%. Therefore, their molecular behaviours are expected to be similar. The malachite assay of four KdsBs was carried out with a home-made chemical library composed of two hundred compounds. All of the chemicals excluding Rose Bengal did not show any inhibitory tendency against four KdsBs. Interestingly, inhibitory activity of Rose Bengal appeared against the three KdsBs (*Bp*KdsB, *Bt*KdsB, and *Pa*KdsB). However, its inhibitory effect is negligible against *Cp*KdsB. *T*_m_ value measurement through CD spectrometry was performed to independently confirm the binding of Rose Bengal with *Bp*KdsB. Intriguingly, a *T*_m_ value of *Bp*KdsB complexed with Rose Bengal could not be obtained because the thermostability of the complex was increased. Therefore, a thermostability test was done using a hit block and proved that *Bp*KdsB bound with Rose Bengal became thermostable at 100 °C for 5 min. In contrast, its native form was precipitated at 80 °C for 5 min. Therefore, the thermostability test could be an independent method to prove the binding capability of chemicals in the case of the KdsB family. The same result was obtained with *Bt*KdsB and *Pa*KdsB.

**Figure 5.**
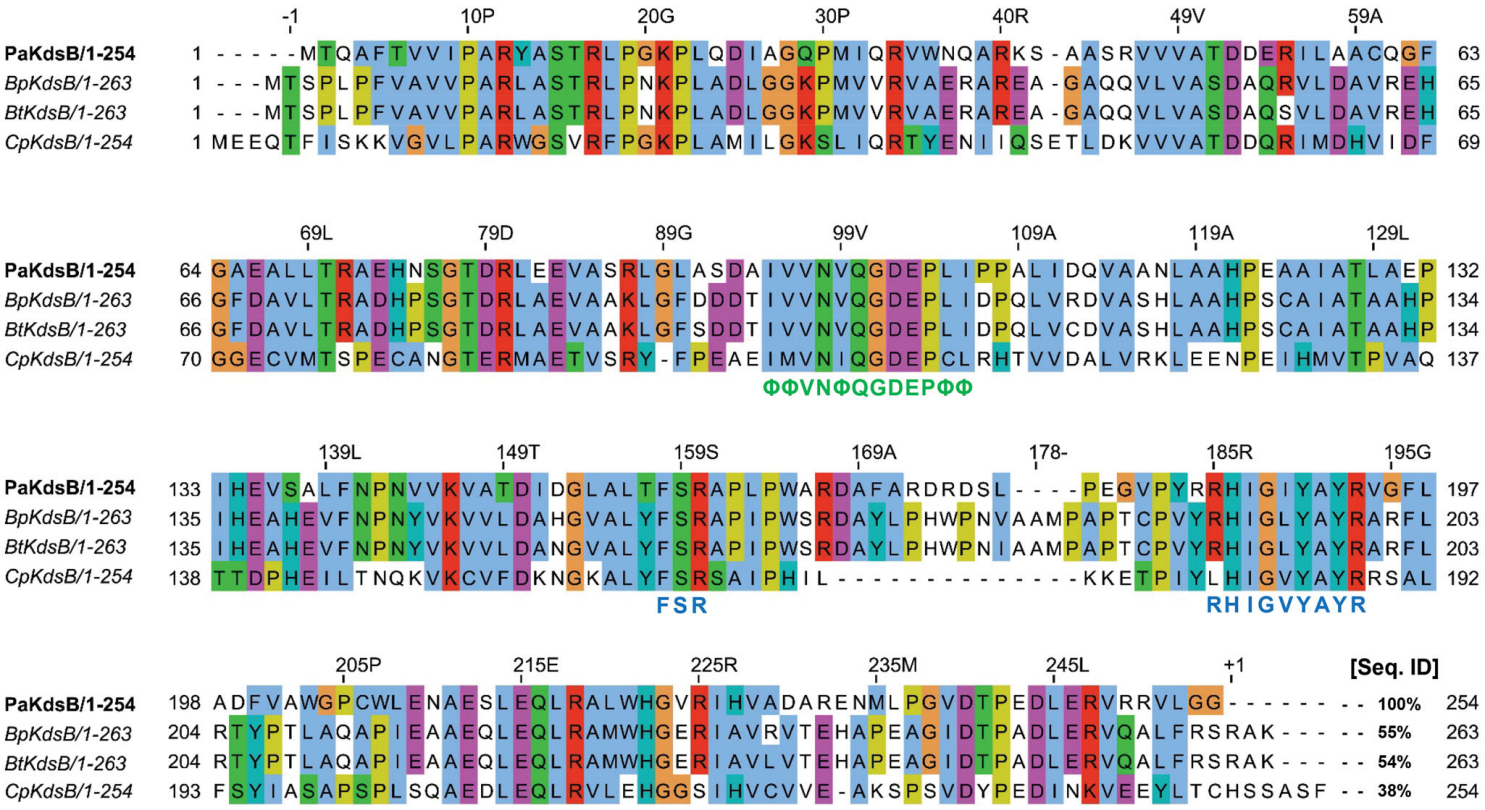
Sequence alignment of *Pa*KdsB with the rest of three KdsBs. *Pa*: *Pseudomonas aeruginosa*; *Bp*: *Burkholderia pseudomallei*; *Bt*: *Burkholderia thailandensis*; *Cp*: *Chlamydia psittaci*. The highly conserved motifs were indicated. Green letters mean the nucleotide binding motif in the catalytic cavity. Blue letters mean the substrate binding motifs. The % sequence identity was calculated with *Pa*KdsB as a reference sequence. The consensus amino acid ID coloured according to the Clustal X colour scheme provided by the Jalview program^37^.

Enzyme kinetics was performed with three KdsBs to identify the inhibitory effect of Rose Bengal (Table 2, Figure 1) and its IC_50_ values (Figure 2). The IC_50_ values are 5.31 µM for *Bp*KdsB, 7.24 µM for *Bt*KdsB, and 14.66 µM for *Pa*KdsB. Therefore, Rose Bengal is a quite good candidate for further development as potential antibiotics. In order to understand the inhibition mechanism of Rose Bengal, the enzyme activity of *Pa*KdsB was determined as a function of KDO concentration in the absence or in the presence of Rose Bengal. The plot obtained reflected typical competitive inhibition kinetics (Figure 3(a)). On the other hand, a non-competitive inhibition mode of binding was exhibited with respect to CTP (Figure 3(b)). The result indicated that Rose Bengal competitively occupies the substrate binding site rather than the nucleotide binding site. For the understanding of the inhibitory activity of Rose Bengal against *Pa*KdsB in the atomic level, an induced-fit molecular docking experiment against the substrate and nucleotide binding sites of *Pa*KdsB using Glide scoring and docking methodology was executed. The Glide *g*-score obtained from the docking on the substrate binding site was –7.19 and that from the nucleotide binding site was –5.62, respectively. The better Glide *g*-score from the substrate binding site implies that the affinity of Rose Bengal is greater at the substrate binding site. In the top model, Rose Bengal is well docked in the substrate pocket of *Pa*KdsB. There are major three interactions between Rose Bengal and *Pa*KdsB (Figure 4). At first, there are two hydrogen bonds between the 6-oxo moiety of Rose Bengal and two residues, Asn98 and Gln100, on the nucleotide binding motif, ^95^ΦΦVNΦQGDEPΦΦ^106^ (Figure 5). Second, there is one salt bridge between the 3-hydroxyl moiety of Rose Bengal and Arg160 on the substrate binding motif, ^158^FSR^160^. Third, there are also one salt bridge and one hydrogen bond between the benzoic acid moiety of Rose Bengal and Arg185 on another substrate binding motif, ^185^RHIGVYAYR^193^. As shown in the sequence alignment, the Rose Bengal binding motifs found in *Pa*KdsB are also conserved in the other two KdsBs. The similar experimental result of the two KdsBs can be explained by the sequence homology. However, *Cp*KdsB shares low sequence homology and some of its residues in the highly conserved motifs are divergent (Figure 5). Specially, the last interaction operated by Arg185 is not possible in the case of *Cp*KdsB. The conserved arginine on this motif is replaced by leucine in *Cp*KdsB. The overall result indicated that Rose Bengal tightly binds to *Bp*KdsB, *Bt*KdsB, and *Pa*KdsB. Considering the increased thermostability of the three KdsBs, the binding of Rose Bengal would trigger a conformational change. The structure of *Pa*KdsB indicates that its valley-like centre is open and lined up by two domains (Figure 4(a)). Rose Bengal seems to induce a closed conformation of *Pa*KdsB, which may confer the resistance of *Pa*KdsB against thermal deactivation.

Rose Bengal is an organic anion dye that has been clinically used for decades and has been used to assess eye surface epithelium damage of eye surface disease^38^. Also, it is a water-soluble photosensitiser with a distinctive wine colour^39^. It is well known that singlet-oxygen production of Rose Bengal after exposure with 300–600 nm radiation increases the antibacterial effect^40–43^. Besides, Rose Bengal was reported as a potent inhibitor of the DNA dependent RNA polymerase of *E. coli* without photooxidation^44^. On the other hand, Rose Bengal can be a possible choice in the treatment of cancer. For instance, PV-10 (injective Rose Bengal developed by Provectus) was found to cause visible therapeutic responses in melanoma patients^45^. Since 2016, PV-10 has been performed as a single treatment for locally advanced cutaneous melanoma patients who participated in phase 3 of clinical research (clinical trials ID NCT02288897). Rose Bengal also has been shown to prevent the growth and spread of ovarian cancer, and to induce the apoptotic cell death of the cancer cells *in vitro*^46^. Therefore, Rose Bengal possesses various beneficial functions against human diseases. This study also suggests a potential usage of Rose Bengal as an antibiotic targeting three KdsBs. Unfortunately, however, the antibiotic effect of Rose Bengal against Gram-negative species was reported to be inefficient due to the presence of LPS obstructing the absorbance of charged compounds^47^^,^^48^. Therefore, photosensitising techniques to induce the antibiotic property of Rose Bengal by producing reactive oxygen species have been employed.

Nevertheless, the discovery of the direct inactivation of three KdsBs by Rose Bengal was informative and thus can be applied to enhance the antibiotic property of Rose Bengal. Specially, we propose the combined usage of Rose Bengal with polymyxin B nonapeptide (PMBN). PMBN has been suggested to increase the permeability of Rose Bengal into OMs of Gram-negative bacteria^49^. Since Rose Bengal-mediated photoinactivation against multidrug resistance *Pa* has been known^50^, a combined therapy increasing absorption of Rose Bengal with antimicrobial peptides should be tested^51^. All the properties of Rose Bengal can be applied and developed to design an anti-melioidosis agent. The crystallisation of the three KdsBs complexed with Rose Bengal is going on to determine the complex structures with X-ray crystallography.

## Conclusions

KdsB is one of the key enzymes in the CMP-KDO biosynthesis pathway. Two researches to develop antibiotics targeting this enzyme were reported. One targeted KdsB from *E. coil*^52^ and the other for *Agrobacterium tumefaciens* and *Aeromonas salmonicida*^53^. However, no further study has been followed. This study discovered the probability of Rose Bengal as a potential antibiotic agent targeting KdsB. Rose Bengal has beneficiary roles in other human diseases and possesses a photodynamic property. Considering a severe fatality of melioidosis caused by *Bp* and multidrug-resistance of *Pa*, the development of Rose Bengal and its derivatives with various combined therapies may provide a promising way to overcome these disease.

## Supplementary Material

Supplemental Material

## References

[1] Smyth KM, Marchant A. Conservation of the 2-keto-3-deoxy-*manno*-octulosonic acid (Kdo) biosynthesis pathway between plants and bacteria. Carbohydr Res 2013;380:70–5. 23974348 10.1016/j.carres.2013.07.006

[2] Mamat U, Schmidt H, Munoz E, et al. WaaA of the hyperthermophilic bacterium *Aquifex aeolicus* is a monofunctional 3-deoxy-d-*manno*-oct-2-ulosonic acid transferase involved in lipopolysaccharide biosynthesis. J Biol Chem 2009;284:22248–62. 19546212 10.1074/jbc.M109.033308 PMC2755949

[3] Hove-Jensen B, Maigaard M. *Escherichia coli* rpiA gene encoding ribose phosphate isomerase A. J Bacteriol 1993;175:5628–35. 8366047 10.1128/jb.175.17.5628-5635.1993 PMC206620

[4] Meredith TC, Woodard RW. *Escherichia coli* YrbH is a d-arabinose 5-phosphate isomerase. J Biol Chem 2003;278:32771–7. 12805358 10.1074/jbc.M303661200

[5] Radaev S, Dastidar P, Patel M, et al. Structure and mechanism of 3-deoxy-d-*manno*-octulosonate 8-phosphate synthase. J Biol Chem 2000;275:9476–84. 10734095 10.1074/jbc.275.13.9476

[6] Park J, Lee D, Kim MS, et al. A preliminary X-ray study of 3-deoxy-d-*manno*-oct-2-ulosonic acid 8-phosphate phosphatase (YrbI) from *Burkholderia pseudomallei*. Acta Crystallogr F Struct Biol Commun 2015;71:790–3. 26057814 10.1107/S2053230X15006135 PMC4461349

[7] Belunis CJ, Raetz CR. Biosynthesis of endotoxins. Purification and catalytic properties of 3-deoxy-d-*manno*-octulosonic acid transferase from *Escherichia coli*. J Biol Chem 1992;267:9988–97. 1577828

[8] Hammond SM, Claesson A, Jansson A, et al. A new class of synthetic antibacterials acting on lipopolysaccharide biosynthesis. Nature 1987;327:730–2. 3037377 10.1038/327730a0

[9] Goldman R, Kohlbrenner W, Lartey P, Pernet A. Antibacterial agents specifically inhibiting lipopolysaccharide synthesis. Nature 1987;329:162–4. 3041230 10.1038/329162a0

[10] Claesson A, Jansson AM, Pring BG, et al. Design and synthesis of peptide derivatives of a 3-deoxy-d-*manno*-2-octulosonic acid (KDO) analogue as novel antibacterial agents acting upon lipopolysaccharide biosynthesis. J Med Chem 1987;30:2309–13. 3681901 10.1021/jm00395a022

[11] Rick D, Osborn MJ. Isolation of a mutant of *Salmonella typhimurium* dependent on d-arabinose-5-phosphate for growth and synthesis of 3-deoxy-d-mannoctulosonate (ketodeoxyoctonate). Proc Natl Acad Sci USA 1972;69:3756–60. 4566459 10.1073/pnas.69.12.3756 PMC389865

[12] Cheng AC, Currie BJ. Melioidosis: epidemiology, pathophysiology, and management. Clin Microbiol Rev 2005;18:383–416. 15831829 10.1128/CMR.18.2.383-416.2005 PMC1082802

[13] Limmathurotsakul D, Peacock SJ. Melioidosis: a clinical overview. Brit Med Bull 2011;99:125–39. 21558159 10.1093/bmb/ldr007

[14] Peacock SJ. Melioidosis. Curr Opin Infect Dis 2006;19:421–8. 16940864 10.1097/01.qco.0000244046.31135.b3

[15] Wiersinga WJ, Currie BJ, Peacock SJ. Melioidosis. N Engl J Med 2012;367:1035–44. 22970946 10.1056/NEJMra1204699

[16] Bartley PP, Pender MP, Woods ML 2nd, Walker D, et al. Spinal cord disease due to melioidosis. Trans R Soc Trop Med Hyg 1999;93:175–6. 10450444 10.1016/s0035-9203(99)90299-7

[17] Caldera AS, Kumanan T, Corea E. A rare cause of septic arthritis: melioidosis. Trop Doct 2013;43:164–6. 24067292 10.1177/0049475513505091

[18] Jane L, Crowe A, Daffy J, Gock H. *Burkholderia pseudomallei* osteomyelitis: an unusual cause of fever in a returned traveller. Aust Med J 2012;5:141–3. 10.4066/AMJ.2012.1025 PMC3413932 22905056

[19] McLeod C, Morris PS, Bauert PA, et al. Clinical presentation and medical management of melioidosis in children: a 24-year prospective study in the Northern Territory of Australia and review of the literature. Clin Infect Dis 2015;60:21–6. 25228703 10.1093/cid/ciu733

[20] Ngamdee W, Tandhavanant S, Wikraiphat C, et al. Competition between *Burkholderia pseudomallei* and *B. thailandensis*. BMC Microbiol 2015;15:56. 25879538 10.1186/s12866-015-0395-7 PMC4365494

[21] Glass MB, Gee JE, Steigerwalt AG, et al. Pneumonia and septicemia caused by *Burkholderia thailandensis* in the United States. J Clin Microbiol 2006;44:4601–4. 17050819 10.1128/JCM.01585-06 PMC1698378

[22] Lertpatanasuwan N, Sermsri K, Petkaseam A, et al. Arabinose-positive *Burkholderia pseudomallei* infection in humans: case report. Clin Infect Dis 1999;28:927–8. 10.1086/517253 10825075

[23] Lyczak JB, Cannon CL, Pier GB. Establishment of *Pseudomonas aeruginosa* infection: lessons from a versatile opportunist. Microbes Infect 2000;2:1051–60. 10967285 10.1016/s1286-4579(00)01259-4

[24] Franzetti F, Cernuschi M, Esposito R, Moroni M. *Pseudomonas* infections in patients with AIDS and AIDS-related complex. J Intern Med 1992;231:437–43. 1588272 10.1111/j.1365-2796.1992.tb00957.x

[25] Kielhofner M, Atmar RL, Hamill RJ, Musher DM. Life-threatening *Pseudomonas aeruginosa* infections in patients with human immunodeficiency virus infection. Clin Infect Dis 1992;14:403–11. 1554824 10.1093/clinids/14.2.403

[26] Bendig JW, Kyle PW, Giangrande PL, et al. Two neutropenic patients with multiple resistant *Pseudomonas aeruginosa* septicaemia treated with ciprofloxacin. J R Soc Med 1987;80:316–7. 3112380 10.1177/014107688708000521 PMC1290820

[27] Ruiz-Garbajosa P, Cantón R. Epidemiology of antibiotic resistance in *Pseudomonas aeruginosa*. Implications for empiric and definitive therapy. Rev Esp Quimioter 2017;30:8–12. 28882007

[28] Boucher HW, Talbot GH, Bradley JS, et al. Bad bugs, no drugs: no ESKAPE! An update from the Infectious Diseases Society of America. Clin Infect Dis 2009;48:1–12. 19035777 10.1086/595011

[29] Michael CA, Dominey-Howes D, Labbate M. The antimicrobial resistance crisis: causes, consequences, and management. Front Public Health 2014;2:145. 25279369 10.3389/fpubh.2014.00145 PMC4165128

[30] Yi H, Kim K, Cho KH, et al. Substrate spectrum extension of PenA in *Burkholderia thailandensis* with a single amino acid deletion, Glu168del. Antimicrob Agents Chemother 2012;56:4005–8. 22564834 10.1128/AAC.00598-12 PMC3393396

[31] Sha S, Zhou Y, Xin Y, Ma Y. Development of a colorimetric assay and kinetic analysis for *Mycobacterium tuberculosis* d-glucose-1-phosphate thymidylyltransferase. J Biomol Screen 2012;17:252–7. 21940712 10.1177/1087057111421373

[32] Kim S, Jo S, Kim M-S, Shin DH. A study of a potent inhibitor against a GDP-6-deoxy-α-d-manno-heptose biosynthesis pathway as antibiotic candidates. Microb Drug Resist 2019; ahead of print. 10.1089/mdr.2019.0144 31613705

[33] Niyamat IB, Suhail AARS, Sandesh RJ, Habib MP. Rose Bengal sensitized niobium pentaoxide photoanode for dye sensitized solar cell application. AIP Conference Proceedings; 2017;1832:040022.

[34] Sherman W, Day T, Jacobson MP, et al. Novel procedure for modeling ligand/receptor induced fit effects. J Med Chem 2006;49:534–53. 16420040 10.1021/jm050540c

[35] Jacobson MP, Pincus DL, Rapp CS, et al. A hierarchical approach to all-atom protein loop prediction. Proteins 2004;55:351–67. 15048827 10.1002/prot.10613

[36] Friesner RA, Murphy RB, Repasky MP, et al. Extra precision glide: docking and scoring incorporating a model of hydrophobic enclosure for protein–ligand complexes. J Med Chem 2006;49:6177–96. 17034125 10.1021/jm051256o

[37] Waterhouse AM, Procter JB, Martin DMA, et al. Jalview Version 2—a multiple sequence alignment editor and analysis workbench. Bioinformatics 2009;25:1189–91. 19151095 10.1093/bioinformatics/btp033 PMC2672624

[38] Kim J. The use of vital dyes in corneal disease. Curr Opin Ophthalmol 2000;11:241–7. 10977768 10.1097/00055735-200008000-00005

[39] Nakonechny F, Barel M, David A, et al. Dark antibacterial activity of Rose Bengal. Int J Mol Sci 2019;20:3196. 10.3390/ijms20133196 PMC6651402 31261890

[40] Ilizirov Y, Formanovsky A, Mikhura I, et al. Effect of photodynamic antibacterial chemotherapy combined with antibiotics on Gram-positive and Gram-negative bacteria. Molecules 2018;23:3152. 10.3390/molecules23123152 PMC6320794 30513653

[41] Schäfer M, Schmitz C, Facius R, et al. Systematic study of parameters influencing the action of Rose Bengal with visible light on bacterial cells: comparison between the biological effect and singlet-oxygen production. Photochem Photobiol 2000;71:514–23. 10818781 10.1562/0031-8655(2000)071<0514:ssopit>2.0.co;2

[42] Nisnevitch M, Nakonechny F, Nitzan Y. Photodynamic antimicrobial chemotherapy by liposome-encapsulated water-soluble photosensitizers. Bioorg Khim 2010;36:396–402. 20644595 10.1134/s106816201003012x

[43] Nitzan Y, Nisnevitch M. Special features of Gram-positive bacterial eradication by photosensitizers. Recent Pat Antiinfect Drug Discov 2013;8:88–99. 23550546 10.2174/1574891x113089990013

[44] Wu FY, Wu CW. Rose Bengal. Inhibitor of ribonucleic acid chain elongation. Biochemistry 1973;12:4343–8. 4584323 10.1021/bi00746a007

[45] Thompson JF, Hersey P, Wachter E. Chemoablation of metastatic melanoma using intralesional Rose Bengal. Melanoma Res 2008;18:405–11. 18830132 10.1097/CMR.0b013e32831328c7

[46] Koevary SB. Selective toxicity of Rose Bengal to ovarian cancer cells in vitro. Int J Physiol Pathophysiol Pharmacol 2012;4:99–107. 22837809 PMC3403562

[47] Fung DYC, Miller RD. Effect of dyes on bacterial growth. Appl Environ Microbiol 1973;25:793–9. 10.1128/am.25.5.793-799.1973 PMC380914 4577179

[48] Koufen P, Zeidler U, Stark G. Photodynamic inactivation of ion channels formed by the polyene antibiotic amphotericin B in lipid membranes. J Photochem Photobiol 1997;38:129–35.

[49] M-Ali HA, Mohammad AS, Majed MM, et al. Modeling the effect of Rose Bengal on growth and decay patterns of *Pseudomonas aeruginosa*, *Escherichia coli* and *Staphylococcus aureus*. IOP Conf Ser Mater Sci Eng 2018;305:012004.

[50] Nakonieczna J, Wolnikowska K, Ogonowska P, et al. Rose Bengal-mediated photoinactivation of multidrug resistant *Pseudomonas aeruginosa* is enhanced in the presence of antimicrobial peptides. Front Microbiol 2018;9:1949. 30177928 10.3389/fmicb.2018.01949 PMC6110182

[51] Olaitan AO, Morand S, Rolain JM. Mechanisms of polymyxin resistance: acquired and intrinsic resistance in bacteria. Front Microbiol 2014;5:643. 25505462 10.3389/fmicb.2014.00643 PMC4244539

[52] Norbeck DW, Rosenbrook W, Kramer JB, et al. A novel prodrug of an impermeant inhibitor of 3-deoxy-d-*manno*-2-octulosonate cytidylyltransferase has antibacterial activity. J Med Chem 1989;32:625–9. 2537425 10.1021/jm00123a021

[53] Goldman RC, Capobianco JO, Doran CC, Matthysse AG. Inhibition of lipopolysaccharide synthesis in *Agrobacterium tumefaciens* and *Aeromonas salmonicida*. J Gen Microbiol 1992;138:1527–33. 1324975 10.1099/00221287-138-7-1527

