# Supplementary material for: A study of Rose Bengal against a 2-keto-3-deoxy-d-manno-octulosonate cytidylyltransferase as an antibiotic candidate
Source: J Enzyme Inhib Med Chem. 2020 Jun 26;35(1):1414–21. doi: 10.1080/14756366.2020.1751150 (PMC7717453; doi:10.1080/14756366.2020.1751150)
Supplement: Supplemental Material [file IENZ_A_1751150_SM4932.pdf]

Journal of Enzyme Inhibition and Medicinal Chemistry

A study of Rose Bengal against a 2-keto-3-deoxy-D-*manno*-octulosonate cytidylyltransferase as an antibiotic candidate

Suwon Kim[1], Seri Jo[1], Mi-sun Kim[1] and Dong Hae Shin*[1]

[1]College of Pharmacy and Graduate School of Pharmaceutical Sciences, Ewha W. University, 52, Ewhayeodae-gil, Seodaemun-gu, Seoul 03760, Republic of Korea

Corresponding author

Prof. Dong Hae Shin

Department of Pharmacy, Ewha W. University, Seoul, 03760, Republic of Korea

Tel: +82-2-3277-4502

Fax: +82-2-3277-2851

E-mail: dhshin55@ewha.ac.kr

Supplementary Table 1. A Chemical Library

No.	Name of compound
1	(-)-Epicatechin
2	(−)-Catechin gallate
3	(−)-Epicatechin
4	(−)-Epigallocatechin gallate
5	(−)-Gallocatechin
6	(−)-Gallocatechin gallate
7	(+)-Catechin hydrate
8	(±)-Catechin
9	(±)-Taxifolin hydrate
10	2',7'-Dichlorofluorescein Sodium
11	2-Pyridinealdoxime methochloride (2-PAM)
12	2,2′,4′-Trihydroxychalcone
13	2,4-Dinitrophenylhydrazine
14	2,4,6-trimethyl-7-oxo-1,3,5-cycloheptatrien-1-yl 4-chlorobenzoate (Chembridge 5945310)
15	3,3',5,5'-Tetramethylbenzidine
16	4-(2-Hydroxyethyl)-1-piperazinepropanesulfonic acid (EPPS)
17	4-Vinylpyridine
18	4,5,6,7-Tetrachloro-2′,4′,5′,7′-tetraiodofluorescein disodium salt (Rose Bengal)
19	5-Azacytidine crystalline
20	5-Nitroindole
21	6-bromochromone-3-carbonitrile
22	6-Diazo-5-oxo-L-norleucine
23	7-Isopropoxy-3-phenyl-4H-1-benzopyran-4-one (Ipriflavone)
24	8-Hydroxyquinoline
25	9-(4'-Dimethylaminophenyl)-2,6,7-trihydroxyfluorone sulfate hydrate
26	9-Aminoacridine hydrochloride
27	Acacetin
28	Acriflavine

29	Actinomycin D from *Streptomyces sp.*
30	Acyclovir
31	Adenosin 5'-(β-γ-imido) triphosphate (AMPPNP)
32	Amentoflavone
33	Amphotericin B
34	Ampicillin
35	Angelicin
36	Antipain
37	Aphidicolin from *Nigrospora sphaerica*
38	Aprotinin
39	Astilbin from Engelhardtia roxburghiana
40	Auraptene
41	Azaserine
42	Bacitracin zinc salt from *Bacillus licheniformis*
43	Baicalein
44	Baicalin
45	Bakuchiol
46	Bavachin
47	beta-Naphthoflavone
48	Borane dimethylamine complex
49	Calcium Ionophore A23187
50	Carbadox
51	Cardamonin
52	Cefazolin
53	Cefoperazone
54	Cefotaxime
55	Chloramphenicol
56	Chloroquine
57	Chlorpromazine
58	Chlortetracycline
59	Cinoxacin
60	Cirsiliol
61	cis-5,8,11,14,17-Eicosapentaenic acid

62	Costunolide
63	Crystal Violet
64	Cycloheximide
65	Cytosine β-D-arabinofuranoside hydrochloride
66	D-(+) Galactosamine
67	D-Fructose 1,6-bisphosphate
68	D-Fructose 6-phosphate
69	D-Ribose 5-phosphate
70	Daidzein
71	Dehydrocostus lactone
72	Dienestrol
73	Dimethylethylammoniumpropane sulfonate (NDSB-195)
74	Dimetridazole
75	Diosmetin
76	Diosmin
77	Docosahexaenoic acid
78	Erythromycin
79	Ethyl 4-cyano-3-methyl-5-({[4- methyl-2- pyrimidinyl)thio]acetyl}-2-thiophenecarboxylate (Chembridge 7698174)
80	Ethyl 4-cyano-3-methyl-5-{[(4H-1,2,4-triazol-3-ylthio)acetyl]amino}-2-thiophenecarboxylate (Chembridge 7570508)
81	ethyl 4-cyano-5-[({[5-(2-ethoxyphenyl)-1,3,4-oxadiazol-2-yl]thio}acetyl)amino]-3-methyl-2-thiophenecarboxylate (Chembridge 7991890)
82	ethyl 4-cyano-5-[({[5-(3,5-dimethoxyphenyl)-1,3,4-oxadiazol-2-yl]thio}acetyl)amino]-3-methyl-2-thiophenecarboxylate (Chembridge 7916711)
83	Ethyl 5-({[(5-benzyl-1,3,4-oxadiazol-2-yl)thio]acetyl}amino)-4-cyano-3-methyl-2-thiophenecarboxylate (Chembridge 7929959)
84	Ethyl 5-[({[5-(4-chlorophenyl)-1,3,4-oxadiazol-2-yl]thio}acetyl)amino]-4-cyano-3- methyl-2-thiophenecarboxylate (Chembridge 7933420)
85	Ethyl acetate
86	Ethylene glycol
87	FCLA Free Acid (Chemiluminescence Reagent)
88	Fisetin
89	Fisetin
90	Flavanone
91	Fluorescein
92	Fusaric acid from *Gibberella fujikuroi*

93	G 418 disulfate
94	Gastrodin
95	Geniposide
96	Geniposidic acid
97	Genistein
98	Genistin
99	Gentamicin
100	Glabridin
101	Guanosine 5′-[β,γ- imido]triphosphate (=GMPPNP)
102	Helichrysetin
103	Herbacetin
104	Hesperidin
105	Hispidulin
106	Homoplantaginin
107	Hygromycin B from *Streptomyces hygroscopicus*
108	Ibuprofen
109	Icaritin
110	Indomethacin
111	Iodoacetic acid
112	Irgasan
113	Isobavachalcone
114	Isoxanthohumol
115	Ivermectin
116	Josamycin
117	Kaempferol
118	Kaempferol 7-O-β -D-glucopyranoside
119	Kanamycin Sulfate
120	L-Ascorbic acid
121	L-Homocitrulline.
122	L-Lysine ethyl ester dihydrochloride
123	Levofloxacin
124	Lincomycin hydrochloride
125	Liriodendrin

126	Luteolin
127	Lysostaphin from *Staphylococcus staphylolyticus*
128	Mangiferin
129	Metronidazole
130	Mevastatin
131	Morin<br>(Morin hydrate)
132	Mycophenolic acid
133	Myricetin
134	N-(6-Aminohexyl)-5-chloro-1-naphthalenesulfonamide Hydrochloride (W-7 Hydrochloride)
135	N-[2-(p-Bromocinnamylamino)ethyl]-5-isoquinolinesulfonamide dihydrochloride<br>(H-89 dihydrochloride)
136	N-Hydroxy-1,8-naphthalimide
137	n-Propyl Gallate
138	Nalidixic acid
139	Naringenin
140	Naringenin
141	Naringin
142	Neohesperidin dihydrochalcone
143	Neomycin
144	Nile Blue A
145	Ochratoxin A from *Petromyces albertensis*
146	Orientin
147	Orotic acid
148	Oroxin B
149	Paclitaxel
150	Pectolinarin
151	Penicillin G
152	Phenazine methosulfate
153	Phenylbutazone
154	Phenylfluorone
155	Pinoresinol diglucoside
156	Polyvinylpyrrolidone
157	Poncirin

158	Praziquantel
159	Prinomastat
160	Prothionamide
161	Puerarin
162	Puromycin dihydrochloride from *Streptomyces alboniger*
163	Pyrantel pamoate
164	Pyrazinecarboxamide
165	Quercetin 3-β-D-glucoside
166	Quercetin hydrate
167	Quercitrin hydrate
168	Raltegravir
169	Rhodamine 6G
170	Rhoifolin<br>(Apigenin 7-O-neohesperidoside)
171	Rifamycin SV sodium
172	Rutin
173	Sakuranetin
174	Saponin
175	Silibinin
176	Silymarin
177	Sinensetin
178	Skullcapflavone II
179	Sodium deoxycholate
180	Sofalcone
181	Sorbic acid
182	Spectinomycin
183	Streptomycin
184	Streptozocin
185	Sulfachloropyridazine
186	Sulfathiazole sodium
187	Tetrabromofluorescein
188	Thiabendazole
189	Triethylamine
190	Trifluoperazine

191	Tylosin
192	Xylene Cyanol FF
193	β-Cyclodextrin
